# Semen Protein CRISP3 Promotes Reproductive Performance of Boars through Immunomodulation

**DOI:** 10.3390/ijms25042264

**Published:** 2024-02-14

**Authors:** Yonghui Bu, Ping Wang, Siqi Li, Li Li, Shouquan Zhang, Hengxi Wei

**Affiliations:** State Key Laboratory of Swine and Poultry Breeding Industry, National Engineering Research Center for Breeding Swine Industry, Guangdong Provincial Key Laboratory of Agro-Animal Genomics and Molecular Breeding, College of Animal Science, South China Agricultural University, Guangdong 510642, China; buyonghui813@163.com (Y.B.);

**Keywords:** CRISP3, boar, fertility, sperm, reproductive tract immunity

## Abstract

Semen proteins play an important role in male reproductive performance and sperm fertilization ability and can be used as potential biomarkers to evaluate male fertility. The role of cysteine-rich secretory protein 3 (CRISP3) in male reproduction remains unknown. This study aimed to investigate the role of CRISP3 in the reproductive performance of boars. Our results showed that the CRISP3 protein content was significantly and positively correlated with boar fertility, sow delivery rate, and litter size. CRISP3 is highly expressed in the bulbourethral gland of adult boars and is enriched in the seminal plasma. It is localized in the post-acrosomal region of the sperm head and migrates to the anterior end of the tail after capacitation. The CRISP3 recombinant protein did not affect sperm motility and cleavage rate, but it significantly downregulated the mRNA expression of inflammatory factors IL-α, IL-1β, and IL-6 and the protein expression of IL-α and IL-6 in lipopolysaccharide (LPS)-induced RAW264.7 cells, indicating that CRISP3 has an immunomodulatory function. In conclusion, our study suggests that semen CRISP3 protein levels positively correlate with reproductive performance, which may be achieved by regulating immune responses in the female reproductive tract.

## 1. Introduction

Semen proteins in sperm or seminal plasma clearly play an important role in regulating sperm functions, such as maintaining sperm motility, assisting sperm capacitation, and sperm–egg binding. These functions are closely related to the reproductive ability of male animals [1,2] and are potential fertility markers for sperm function and fertility [3]. With the rapid development of proteomics, using biomarkers to screen boars for high reproductive performance [4] is of great significance for improving the production level of animal husbandry.

Cysteine-rich secreted proteins (CRISPs) are an important family of proteins found in the semen of mammals [5,6,7,8]. CRISPs are a branch of the CRISP, Antigen5, and Pathogenesis-related Protein1 superfamily of proteins [9]. Four CRISP proteins have been identified in mice and three in humans, horses, and pigs, and they are highly enriched in the male reproductive tract [10]. Recent studies have shown that the CRISP1 protein can regulate Ca^2+^ channels, and that it plays an important role in preventing sperm hyperactivation [11,12]. An abnormal expression of CRISP2 is associated with fertility problems in humans, and its expression is significantly lower in patients with azoospermia or asthenospermia than in normal fertile men [13,14]. Moreover, recent studies have shown that the expression level of CRISP3 is related to male infertility [13]. Sperms lacking the CRISP4 protein severely affected the fertilization ability of cumulus–oocyte complexes, oocytes with intact zona pellucida, and oocytes without zona pellucida in vitro [15]. The deletion of all four CRISP genes in mice resulted in severe subfertility [16].

The CRISP3 protein was first identified in mouse salivary glands [17] and human neutrophils [18] and is the least-studied member of the CRISP family. Unlike other members, CRISP3 is widely expressed in exocrine organs [19], immune organs, and, to a lesser extent, reproductive organs [20,21]. Studies have shown that human CRISP3 is not involved in gamete fusion [22]. The CRISP3 gene knockout mice showed that, compared to normal mice, there was no significant difference in the fertilization rate of the knockout mice, but the blastocyst rate and the number of pups were significantly reduced [17] CRISP3 protein is involved in the immune response and has a defensive role [23], which can inhibit the binding of sperm to polymorphonuclear neutrophils and prevent sperm from being cleared in the female reproductive tract [24]. We analyzed the differentially expressed proteins in the sperm of boars with high and low fertility using iTRAQ technology [25] and found that CRISP3 was highly expressed in the semen of boars with high fertility. Consistent with previous reports, CRISP3 protein may be related to the reproductive performance of boars.

This study revealed a relationship between the expression level of CRISP3 protein in semen and the reproductive performance of boars. The expression of CRISP3 protein and CRISP3 antibody in sperm and reproductive tissues and the possible mechanism of its function in sperm fertilization was analyzed. This study enriches the expression profile of the CRISP3 protein, provides new ideas for its function in the process of pig reproduction and provides new evidence for CRISP3 as a potential biomarker of male fertility.

## 2. Results

### 2.1. Correlation between Sperm CRISP3 Protein Levels and Reproductive Parameters in Boars

**Figure 1:**
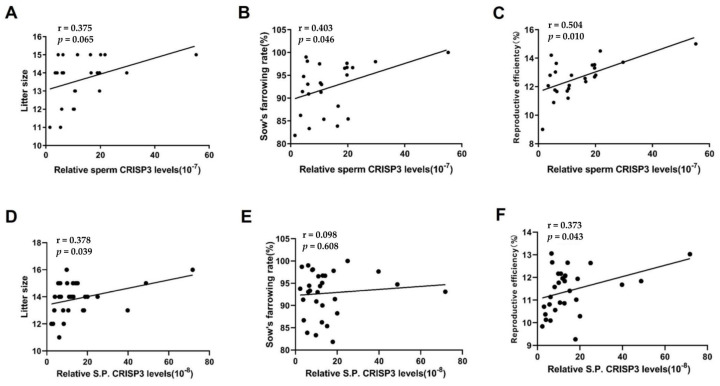
Correlation analysis between the content of CRISP3 and the boar reproductive parameters. Reproductive efficiency equals litter size multiplied by the sow’s farrowing rate. For sperm CRISP3, 25 boars inseminated 1324 sows (**A**–**C**); for seminal plasma CRISP3, 30 boars inseminated 1779 sows (**D**–**F**).

The total protein concentration in the semen or seminal plasma of 33 boars was determined using the BCA method, while the CRISP3 protein concentration was measured through ELISA, and its relative concentration was calculated. After excluding outliers, the relative CRISP3 protein content of the sperm of 25 boars and the seminal plasma of 30 boars was finally obtained. Scatter plots of CRISP3 protein relative content data are represented in Figure S1. The results showed that the sperm CRISP3 protein level was significantly positively correlated with the sow’s farrowing rate and reproductive efficiency (*p* < 0.05). The CRISP3 protein level in seminal plasma was significantly positively correlated with litter size and reproductive efficiency (*p* < 0.05) (Figure 1).

### 2.2. Expression, Purification, and Identification of Recombinant CRISP3 Protein and Antibody

Obtaining an active CRISP3 protein is a key step in investigating the specific mechanisms by which CRISP3 proteins regulate reproductive performance. CRISP3 protein was expressed in CHO-K1 cells, and the supernatant and CHO-K1 cells were collected for identification (Figure S2) and purified using HisTrap HP column tag affinity chromatography. The purified recombinant CRISP3 protein exhibits a molecular weight of 28 kDa with the predicted value, indicating successful expression. To conduct a more comprehensive analysis of the recombinant CRISP3 protein, the purified peptide underwent LC-MS/MS analysis. The amino acid sequence of CRISP3 was determined using LC-MS/MS analysis and subsequently compared with the NCBI database (Accession No. XP_003128480.1). The resulting peptide coverage exceeded 70% (Figure S3).

BALB/C mice were immunized with the recombinant protein CRISP3. Through cell fusion, hybridoma cell screening, subcloning, and ascites preparation and purification, CRISP3 monoclonal antibodies were finally obtained. The heavy and light chains of CRISP3 were 55 and 25 KDa, respectively, on SDS-PAGE, which were consistent with the characteristics of the antibody (Figure S4). The purified antibody concentration was 2.0 mg/mL.

The specificity of the CRISP3 antibody was identified by using the testis and the bulbourethral gland tissues of boars. The amino acid homology of porcine CRISP1, CRISP2, and CRISP3 was also compared. The results showed that the amino acid identity of porcine CRISP1 with CRISP2 and CRISP3 was 42%, and the amino acid identity of CRISP2 and CRISP3 was 63%. The results of the Western blot showed that the CRISP3 antibody could recognize the CRISP3 protein in boar testis and bulbourethral gland tissue, while the CRISP2 antibody could only recognize the target protein in boar testis tissue but not in the bulbourethral gland tissue. Since the anti-CRISP3 antibody also recognizes a band of the same molecular weight as the band recognized by the anti-CRISP2 antibody in the testis, a cross-reaction between them cannot be completely excluded (Figure S5).

To verify whether CRISP3 recombinant proteins have glycosylation modifications, CRISP3 recombinant proteins were treated with glycosidase F for 20 h at 37 °C and subjected to Western blot analysis. The results showed that, compared with the CRISP3 recombinant protein without glycosidase F treatment, the gray value of the CRISP3 recombinant protein treated with glycosidase F increased at 21 KDa and decreased at 29 KDa, the total gray value was similar (Figure S6). These results indicate that CRISP3 recombinant proteins have glycosylation modifications.

### 2.3. Expression and Localization of CRISP3 in Porcine Reproductive Organs and Semen

**Figure 2:**
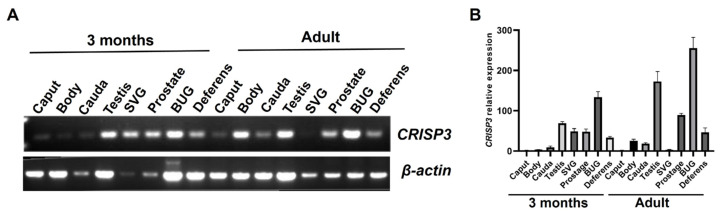
The expression of the CRISP3 gene in the reproductive tissues or cells of boars of different ages. (**A**) Reverse transcriptase PCR detection of CRISP3 gene expression in the boar reproduction systems. (**B**) qRT-PCR detection of CRISP3 gene expression in male reproductive tissues of adult and 3-month-old pigs.

To investigate the potential involvement of the CRISP3 protein in boar fertilization capacity and reproductive capability, we conducted an analysis of CRISP3 gene expression in the reproductive organs of boars using the RT-PCR technique. According to Figure 2A, CRISP3 expression was observed in the testis, epididymis, seminal vesicle, prostate gland, bulbourethral glands, and vas deferens of adult and 3-month-old boars.

Following that, the expression level of CRISP3 was subsequently validated. qRT-PCR was employed to determine the relative expression levels of CRISP3 in various reproductive organs of adult and 3-month-old pigs, including the testis, epididymis, seminal vesicle, prostate gland, bulbourethral glands, and vas deferens. The findings of this study indicate that CRISP3 was highly expressed in the testis, prostate gland, bulbourethral gland, and vas deferens of adult boars, and in the testis, seminal vesicle, prostate gland, bulbourethral glands, and vas deferens of 3-month-old boars. Notably, Figure 2B demonstrates its highest expression in the bulbourethral glands of adult boars.

**Figure 3:**
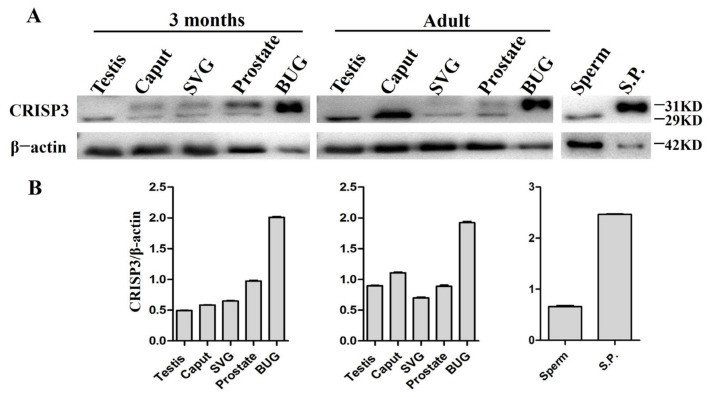
The expression of CRISP3 protein in the reproductive tissues or cells of male pigs with different ages. (**A**) Western blot analysis of CRISP3 protein expression in adult and 3-month-old male reproductive tissues. SVG, seminal vesicle gland; BUG, bulbourethral gland; S.P., seminal plasma; (**B**) Western blot analysis of CRISP3 protein expression in adult and 3-month-old boars’ reproductive tissues using Image J software.

The Western blotting technique was utilized to determine the relative abundance of CRISP3 protein in the reproductive organs and semen of boars across different age groups. The study findings revealed that CRISP3 protein exhibited expression in various adult and 3-month-old male pig reproductive organs, including the testis, epididymis, seminal vesicle gland, prostate, and bulbourethral glands. Notably, a particularly elevated expression level of CRISP3 was observed in the bulbourethral glands. In addition, we found that, in the 3 months of boar epididymis, seminal vesicle and prostate gland, and seminal vesicle gland and prostate of adult boar, CRISP3 protein has two kinds of forms, namely, glycosylated and non-glycosylated forms. CRISP3 was found in a non-glycosylated form in 3-month-old boar testis and adult boar testis, epididymis, and sperm, while it was found in a glycosylated form in the bulbourethral glands and seminal plasma, as illustrated in Figure 3. The obtained results exhibited congruity with our prior research outcomes.

**Figure 4:**
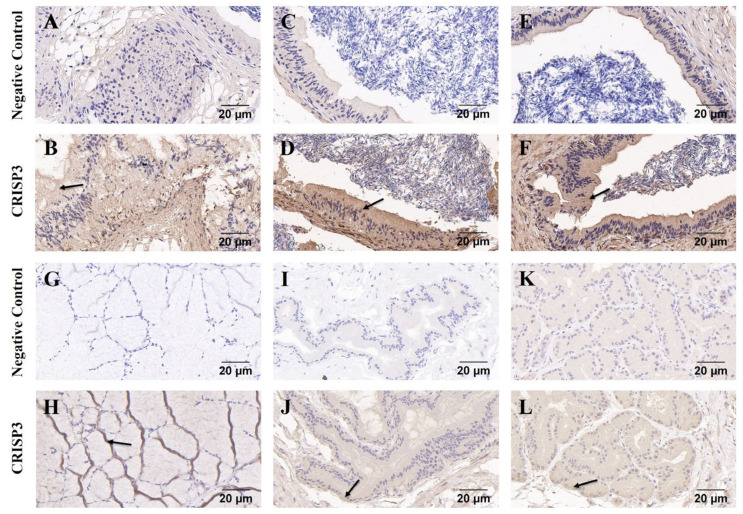
Immunohistochemical analysis of CRISP3 in epididymis and accessory gonads of adult boars. (**A**–**F**) Immunohistochemical analysis of the head, body, and tail of the epididymis of adult boars. (**G**–**L**) Immunohistochemical analysis of bulbourethral gland, seminal vesicle gland, and prostate gland in adult boars, respectively.

Immunohistochemistry was used to detect the distribution of the CRISP3 in the epididymis, prostate, seminal vesicle gland, and bulbourethral glands of adult boars. The findings indicated that the expression of CRISP3 was observed in the cytoplasm of luminal gland epithelial cells in the head, body, and tail of the epididymis and accessory gonads, as compared to the negative control group (Figure 4).

**Figure 5:**
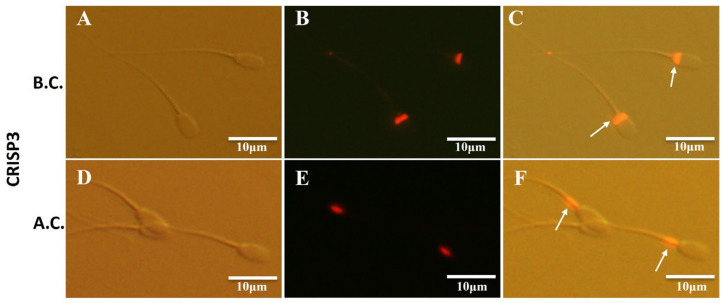
Immunofluorescent staining of CRISP3 in sperm before and after capacitation. (**A**–**C**) Representative images of the immunofluorescent staining of CRISP3 in sperm before capacitation; (**A**) images taken under light microscope, (**B**) images taken under fluorescent scope; (**C**) merged images. (**D**–**F**) Representative images of the immunofluorescent staining of CRISP3 in sperm after capacitation; (**D**) images taken under light microscope, (**E**) images taken under fluorescent scope; (**F**) merged images. B.C., before capacitation; A.C., after capacitation. The arrow indicates the distribution of the target proteins.

The localization of the CRISP3 in sperm was examined using immunofluorescence techniques both before and following sperm capacitation. The results revealed that CRISP3 was mostly present in the post-acrosomal region of the sperm head prior to capacitation. However, after capacitation, CRISP3 was shown to relocalize to the mid-upper portion of the sperm tail (Figure 5). The complete picture is shown in Figure S7.

### 2.4. The Effect of CRISP3 Protein on Sperm Function and Fertilization Ability

The present study aimed to evaluate the impact of the CRISP3 protein on sperm function and its ability to fertilize. There were no statistically significant variations seen in sperm motility and other motility parameters between the group that received CRISP3 antibody treatment for a duration of 30 min and the negative control group (*p* > 0.05) (Table 1).

To investigate the effect of the CRISP3 recombinant protein on sperm mitochondrial function-related genes, the relative mRNA expression levels of COX5B, ATP5F1, UQCRC1, CYC1, and NDUFS8 genes in sperm were detected after incubation with 3, 5, 10, and 20 μg/mL CRISP3 recombinant proteins in semen for 4 h. The results showed that there was no significant difference in the mRNA expression of COX5B, ATP5F1, UQCRC1, CYC1, and NDUFS8 genes between the sperm treated with 3, 5, 10, and 20 μg/mL CRISP3 recombinant proteins and the blank control group (Figure S8).

**Figure 6:**
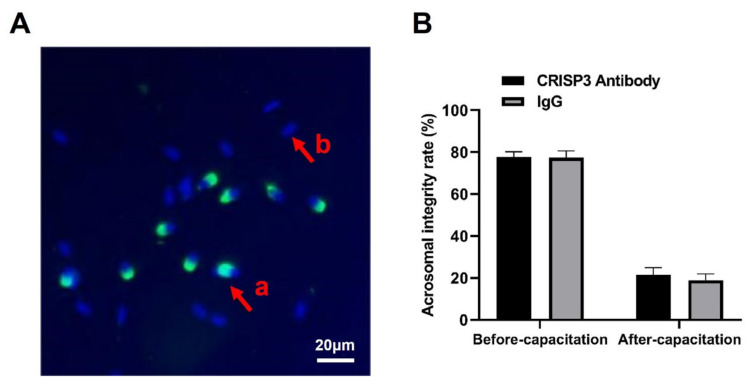
Effects of the CRISP3 antibody on the rate of acrosomal integrity after sperm capacitation in vitro. (**A**) Sperm in different statuses. Sperm samples were fixed with FITC-PNA and Hoechst33342 and scored according to the state of the acrosome. a: sperm has not experienced an acrosome reaction, and b: indicates that an acrosome reaction has occurred. (**B**) Analysis of sperm acrosome reaction rate before and after capacitation with CRISP3 antibody. The data are presented as the mean ± SEM, *n* = 3.

The addition of the CRISP3 antibody to sperm during in vitro capacitation showed no significant difference in the acrosomal integrity rate of sperm before and after capacitation compared with the negative control IgG (*p* > 0.05) (Figure 6).

During the process of IVF, the inclusion of the CRISP3 antibody in the fertilization medium allowed for the subsequent assessment of the embryo cleavage rate after 48 h. The findings indicated that there were no statistically significant disparities in the rate of cleavage among the anti-CRISP3 group (55.04 ± 0.89%), control group (59.53 ± 2.54%), and IgG group (57.50 ± 2.03%) (*p* > 0.05) (Table 2).

### 2.5. CRISP3 Protein Inhibited LPS-Induced Production of Inflammatory Factors in RAW264.7 Cells

**Figure 7:**
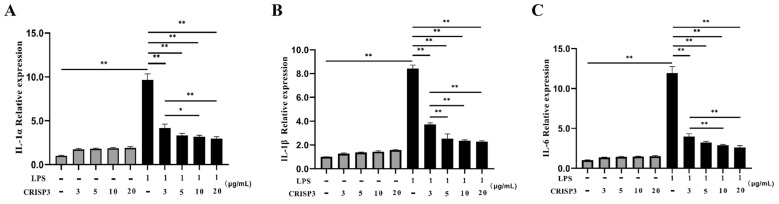
Effect of CRISP3 protein on the mRNA expression levels of inflammatory cytokines IL-1α, IL-1β, and IL-6 in RAW264.7 cells induced by LPS. (**A**) IL-1α mRNA expression level; (**B**) IL-1β mRNA expression level; (**C**) IL-6 mRNA expression level. GAPDH was included in the standardization of all samples as an internal reference gene, and the data were expressed as the mean ± SEM of three independent experiments, * *p* < 0.05, ** *p* < 0.01.

To verify the immunoactivity of the CRISP3 protein and explore the effect of the CRISP3 protein on the expression of intracellular inflammatory factors, we used LPS to induce RAW264.7 cells to establish an inflammation model, and different concentrations of CRISP3 protein were incubated with RAW264.7 cells in the presence or absence of LPS for 6 h. The results demonstrated that different concentrations of CRISP3 protein did not affect inflammatory factor mRNA levels without LPS (*p* > 0.05). Nevertheless, when exposed to LPS, there was a notable augmentation in the relative mRNA expression levels of IL-6, IL-1α, and IL-1β. Pre-incubation with 3, 5, 10, and 20 μg/mL CRISP3 proteins for 30 min significantly decreased the relative mRNA expression levels of IL-6, IL-1α, and Il-1β. In comparison to the control group that did not receive any treatment, there was a notable reduction in the mRNA expression levels of IL-6, IL-1α, and IL-1β (*p* < 0.01) (Figure 7).

**Figure 8:**
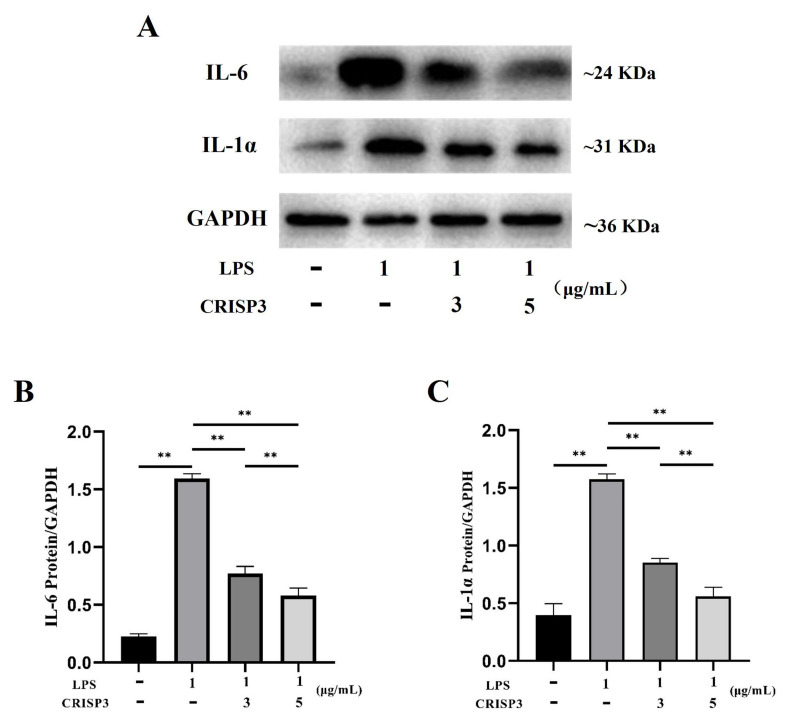
Effect of CRISP3 on the expression levels of IL-1α and IL-6 proteins in RAW264.7 cells induced by LPS. (**A**) Western blot analysis of IL-1α and IL-6 protein expression levels in RAW264.7 cells. (**B**) Western blot analysis of IL-6 protein expression level by Image J. (**C**) Western blot analysis of IL-1α protein expression level by Western blot. Normalized by GAPDH, the data were represented by the mean of three independent experiments ± SEM, ** *p* < 0.01.

The western blot analysis revealed that the application of recombinant CRISP3 at concentrations of 3 and 5 μg/mL resulted in a significant decrease in the expression levels of the inflammatory components IL-6 and IL-1α protein, which were triggered by LPS (Figure 8).

## 3. Discussion

In this study, we analyzed, for the first time, the correlation between the relative content of the sperm protein CRISP3 and the reproductive parameters of boars. We found that the semen CRISP3 protein level was significantly positively correlated with the fertility of boars, sow delivery rate, and litter size. Monoclonal antibodies against CRISP3 were prepared to reveal the expression and localization of CRISP3 in reproductive organs and sperm at the gene and protein levels, which did not affect the fertilization ability of the sperm. The immunoregulatory function of CRISP3 was confirmed in an inflammatory model using LPS-induced RAW264.7 cells.

Usuga et al. found high concentrations of CRISP3 in horse semen, and the concentration in the semen was positively correlated with the pregnancy rate in the first cycle after artificial insemination [26,27]. Similar findings have been reported by Doty et al. CRISP3 appears to attach to the sperm surface and is positively correlated with fertility [9,28], which is consistent with the observed patterns. Sperm proteins are synthesized and secreted by the testes, epididymis, and accessory gonads. Proteins in semen are assembled or bound to the surface of the sperm during sperm formation, maturation, or fertilization and play a role in the fertilization process. It has been reported that CRISP3 is expressed in the testis and epididymis of humans and through the accessory gonads of mice, but not in the epididymis of rats [7,23]. In this study, the reproductive organs of male and female sows of different ages were selected to explore the distribution of CRISP3 in the reproductive systems of male and female sows at both the gene and protein levels. CRISP3 was expressed in the testis, prostate, and bulbourethral glands of adult and 3-month-old male pigs and was enriched in sperm and semen, consistent with the results of Song et al. [29]. We found that the expression of CRISP3 in the bulbourethral glands of adult and 3-month-old male pigs was higher than that in the other tissues. It was previously found that CRISP3 has two isoforms in human and horse semen [23,28,30], 29 kDa and 31 kDa, respectively, depending on its glycosylation status [7]. The present study revealed two forms of CRISP3 in the epididymis, seminal vesicles, and prostate glands. In the testes and sperm, CRISP3 is non-glycosylated, whereas in the bulbourethral glands and semen, CRISP3 is glycosylated. Indirect immunofluorescence localized CRISP3 to the acrosome and tail of human-capacitated sperm [23,31]. Our results showed that CRISP3 was mainly present in the post-acrosome region of the sperm head before capacitation, and CRISP3 was shown to relocalize to the mid-upper part of the sperm tail after capacitation. The specific mechanism of this phenomenon may be that the CRISP3 protein is localized on the surface of the head and migrates to the tail with capacitation, or it is lost from the head and exposed to the tail with capacitation.

Our results showed that sperm CRISP3 was positively correlated with boar fertility, sow delivery rate, and litter size. However, the addition of the CRISP3 antibody to sperm had no significant effect on sperm motility or other parameters. In addition, CRISP3 antibodies do not affect cleavage rates, which has also been demonstrated in the description of human CRISP3 proteins [23]. Double knockout of CRISP1 and CRISP3 in male mice showed a significant reduction in litter size after mating compared to the control group. However, there was no difference in the number of fertilized eggs recovered from the fallopian tube compared to the control group [16]. The positive effects of CRISP3 on the boars’ reproductive ability may be achieved by regulating other reproductive processes. CRISP3 is expressed in reproductive and immune organs, such as the thymus and spleen, which have innated immune functions [32]. Lipopolysaccharide, as a component of gram-negative bacteria, can induce a series of harmful inflammatory responses [33]. RAW264.7 cells are an immortalized mouse macrophage cell line that has been widely used in vitro, and LPS can induce macrophages to release IL-1, IL-6, and other potent inflammatory factors [34]. Therefore, we use a macrophage model to assess the impact of pig CRISP3 recombinant protein on the inflammation induced by LPS. The results showed that the CRISP3 recombinant protein could significantly reduce the LPS-induced expression of IL-1 and IL-6, which proved its immunosuppressive activity. Examination of the sperm in the uterus of female mice after mating with the male CRISP1 and CRISP3 double knockout models showed that the sperm in the uterus of female mice mated with the control group moved freely in the uterine fluid, whereas most of the sperm in the double knockout model group did not move and showed a clustering phenomenon [17] due to the lack of CRISP3 in the seminal plasma. Defects in uterine sperm motility may eventually lead to delayed fertilization and defects in embryonic development [32]. CRISP3 is present in equine seminal plasma and protects sperm from smooth passage through the female reproductive tract by inhibiting neutrophil-mediated phagocytosis of sperm in utero [9,28]. Therefore, CRISP3 may positively affect the reproductive ability of boars by regulating the immune function of the female reproductive tract.

This study examined the effect of CRISP3 recombinant protein on a mouse macrophage cell line in vitro. In the future, systemic studies using CRISP3 should be considered to further verify the effect of CRISP3 on female reproductive tract immunity.

## 4. Materials and Methods

### 4.1. Animals and Samples

Boar tissue samples and semen were collected from the Guangdong Wenshi Shuitai Breeding Farm, and production data for each boar were obtained. The breed of boars and mating sows is Large White. AI was conducted on a single farm with a single dose of 4-billion sperm, and the insemination method was cervical intraoral insemination. Only semen that conforms to the appearance and smell of normal sperm, motility of more than 70%, abnormal rate of less than 20%, and 80 mL of semen containing at least 4-billion sperm are used for AI and other analysis. Sperm and seminal plasma were extracted immediately after semen collection, and tissue samples were taken immediately after slaughter and kept in liquid nitrogen. The male BALB/C mice, aged 6–8 weeks, were procured from the Guangdong Medical Laboratory Animal Center, and ovaries from female pigs were obtained from a nearby abattoir.

### 4.2. Extraction of Protein and Nucleic Acid

As per the guidelines provided by the manufacturer, the Whole Protein Extraction Kit (KeyGen Biotech, Nanjing, China) was used to extract total protein from sperm and tissue samples, and part of the tissue was cut and ground, or the semen was centrifuged at 10,000 rpm for 5 min. After collecting the supernatant, the precipitate was washed 2–3 times with precooled Dulbecco’s Phosphate-Buffered Saline (DPBS). The precipitate was resuspended in lysis buffer containing phosphatase inhibitors, PMSF, and protease inhibitors. After 30 s of swirling and 4 min on ice, the supernatant was centrifuged at 12,000 rpm (4 °C) for 5 min. The supernatant was added to precooled acetone in the ratio 1:2, placed at –20 °C for 4 h, 20 min of centrifugation at 12,000 rpm followed by the discarding of the supernatant and a brief period on ice to collect the precipitate.

RNA was isolated from all samples with an RNeasy Mini Kit (Qiagen, Hilden, Germany). The measurement of RNA purity and concentration was conducted using a NanoDrop ND-1000 instrument (Thermo Fisher Scientific, Waltham, MA, USA). The assessment of RNA integrity was conducted utilizing an Agilent 2100 Bioanalyzer. (Agilent, San Jose, CA, USA).

The quantification of protein levels was conducted utilizing a Bicinchoninic Acid (BCA) Protein Assay Kit (KeyGen Biotech, Nanjing, China). In brief, the BCA working solution was configured according to proportion. Eight concentrations of protein standard solutions were serially diluted with deionized water to establish the standard curve (protein concentration range 0–2000 μg/mL). The total volume of tested samples was 20 μL. All samples and standards were repeated three times, and 200 μL BCA working solution was added. The measurement of absorbance was conducted using colorimetry at a specific wavelength of 562 nm, and, subsequently, a standard curve was constructed. The corresponding protein content (μg/μL) was determined using a standard curve.

### 4.3. Detection of Semen CRISP3 by Enzyme-Linked Immunosorbent Assay (ELISA) and Analysis of Its Correlation with Reproduction

The content of CRISP3 in the sperm and seminal plasma of boars was detected using a CRISP3 ELISA Kit (PG1898, TSZ, USA), following the instructions provided by the manufacturer. All samples, standards, and blank controls were analyzed thrice, and the detection range was 12–1200 pg/mL. The relative concentration of CRISP3 was assessed by calculating the ratio of CRISP3 protein concentration in sperm and seminal plasma to their respective total protein concentrations. Employ Pearson’s correlation analysis to examine the relationship between the relative concentrations of CRISP3 protein in sperm and seminal plasma, and the production data of sows involved in mating.

### 4.4. Preparation of CRISP3 Protein and Antibody

The cDNA of porcine CRISP3 was acquired from the bulbourethral gland tissue of pigs. Based on the genomic DNA sequence of CRISP3 (GenBank accession No. NC_010449.5), the amplified fragment of CRISP3 containing the restriction site was obtained by utilizing forward and reverse primers (5′-GGTGAATTCGCCACCATGGAGACCGACACCCTG-3′ and 5′-ATCGGATCCTCAATGATGATGATGATGATGGTAG-3′, respectively), which was subsequently inserted into the EcoR I and BamH I sites of pLVX. The recombinant plasmid was introduced into competent *Escherichia coli* (*E. coli*) JM109 cells via transfection. Single colonies were established in bacteriolytic broth (LB) containing 100 μg/mL ampicillin at 37 °C and incubated with shaking at 200 rpm/min. The positive clones identified by enzymatic digestion were verified by sequencing. After comparing the sequencing results, the plasmids were extracted.

The HEK293T and CHO-K1 cell lines were inoculated into a 10 cm cell culture dish with a cell density of 1 × 10^5^ cells/mL. The cells were then grown in Dulbecco’s Modified Eagle Medium (DMEM) supplemented with 10% fetal bovine serum (FBS) and maintained in an environment with 5% CO_2_ at a temperature of 37 °C until the cell confluence reached 60%. The HEK293T cells were transfected with recombinant pLVX-CRISP3 and pLVX vector plasmids using LentiFit (HanBio, Shanghai, China), whereas the negative control consisted of the empty vector. After 48 h, the virus solution was collected, concentrated, and used to infect CHO-K1 cells. After selection with puromycin (Thermo Fisher Scientific, USA), the supernatant of CHO-K1 cells was collected and loaded onto a HisTrap HP column (Cytiva, Marlborough, MA, USA). The CRISP3 protein was purified in accordance with the guidelines (HisTrap HP GEexpress Kit manual) provided by the manufacturer and subsequently subjected to dialysis using a phosphate buffer solution (PBS). The purity of the sample was assessed using sodium dodecyl sulfate-polyacrylamide gel electrophoresis (SDS-PAGE), while the sample identification was achieved through liquid chromatography-tandem mass spectrometry (LC-MS/MS). The recombinant CRISP3 protein was stored at −80 °C until its utilization.

Mouse monoclonal antibodies against recombinant CRISP3 protein were prepared according to a previous method [28]. In brief, a total of three administrations were performed every 2 weeks in five BALB/C mice, with each administration consisting of inoculation with 100 µg recombinant CRISP3 protein. Serum was collected from anesthetized mice according to animal welfare standards, and serum titers were determined using ELISA. Based on the serum antibody titer test results, spleen cells from suitable mice were selected for cell fusion with sp2/0 cells, followed by screening and subcloning of hybridoma cells, and, finally, preparation of ascites. Affinity-purified monoclonal antibodies (immunoglobulin G [IgG] fraction) were used as described previously [35]. Purified antibodies were stored in a buffer (50% glycerol, 0.1% sodium azide, 0.1% gelatin) at −80 °C until its utilization.

### 4.5. Reverse Transcriptase PCR and Quantitative Real-Time PCR (qRT-PCR)

RT-PCR was used to find CRISP3 expression in the various reproductive organs of male and female pigs of various ages. The target gene sequence was queried in the National Center for Biotechnology Information (NCBI) (GenBank accession No. NC_010449.5) database, and, subsequently, the primers were generated. The sequences are presented in Table S1. The protocol for the PCR was conducted as follows: an initial pre-denaturation step at a temperature of 95 °C for a duration of 3 min, followed by 35 cycles of denaturation at 95 °C for 15 s, annealing at 58 °C for 15 s, and extension at 72 °C for 60 s, followed by a complete extension at 72 °C for 5 min. The expression of the CRISP3 gene in male and female pig reproductive organs at different ages, as well as IL-1β, IL-1α, and IL-6 in RAW264.7 cells, was confirmed using qRT-PCR. The SYBR qPCR Kit (Vazyme, Nanjing, China) was employed, with GAPDH utilized as the internal reference control. Each sample underwent three replicates. The reaction protocol involved pre-denaturation at 95 °C for 3 min, 40 cycles of denaturation at 95 °C for 10 s, annealing at 60 °C for 30 s, and melting curve acquisition at 95 °C, 60 °C, and 95 °C for 15 s. It was done on an Applied Biosystems 7900HT Real-Time PCR Thermal Cycler. (Applied Biosystems, Foster City, CA, USA).

### 4.6. Western Blotting

The reproductive organ tissues of boars from various age groups, the proteins present in adult boar semen, and the proteins from RAW264.7 cells were isolated using a 12% SDS-PAGE and subsequently transferred onto polyvinylidene difluoride (PVDF) membranes (Millipore, Burlington, MA, USA). After 1 h of blocking with 5% skim milk powder at room temperature, PVDF membranes were washed with TBST (137 mM NaCl, 20 mM Tris, 0.1% Tween-20, pH 7.6) and incubated with primary antibodies overnight at 4 °C. Anti-CRISP3, Anti-CRISP2 antibody (1:2000, SAB2501636, Sigma, Marlborough, MA, USA), IL-1α, and IL-6 (1:1000, Abcam, Waltham, MA, USA) and control antibodies β-actin and GAPDH (1:2000, TransGen, Beijing, China) were employed. After washing the membranes, Goat Anti-Rabbit IgG (H + L) Horseradish Peroxidase (HRP)or Goat Anti-Mouse IgG (H + L) HRP (1:2000, Thermo Fisher Scientific, Waltham, MA, USA) were incubated for 1 h. Automatic imaging equipment (Tanon5200, Shanghai, China) was used to image the membranes after washing and incubating them with chemiluminescence solution in a dark room for 2 min. The experiment was repeated at least three times. The Image J software (https://imagej.nih.gov/ij/index.html, accessed on 23 January 2024) was utilized for the comparative analysis of protein expression levels.

### 4.7. Immunohistochemistry

Freshly collected urethral bulbar glands from adult boars were fixed in paraformaldehyde for 48 h and embedded in paraffin. The samples were subjected to immunohistochemistry using paraffin sections. In short, 5 μm tissue sections were mounted on silicated slides for analysis; the paraffin sections underwent deparaffinization using xylene and subsequent rehydration through a series of graded ethanol solutions. The sections were boiled in 10 mM citric acid (pH 6.0) and incubated in 3% H_2_O_2_ at 26 °C for 25 min in the dark. Three percent bovine serum albumin (BSA) was equally applied to the tissues and blocked at room temperature for one hour. Next, an anti-CRISP3 antibody (1:1000) was incubated overnight at 4 °C. After three PBS washes, the slides were incubated with Goat Anti-Mouse IgG (H + L) HRP (1:2000, Thermo Fisher Scientific, USA) at 37 °C for 1 h. Fresh DAB chromophobe solution was then added. Slides were counterstained with hematoxylin and examined with an Olympus BX53F microscope (Olympus, Tokyo, Japan).

### 4.8. Immunofluorescence Staining

Before and after capacitation in vitro, the sperm density was adjusted and evenly coated on the slide, air-dried for 50 min, fixed in 4% paraformaldehyde for 1 h, rinsed with PBS, blocked with 10% goat serum at 37 °C for 1 h, and then treated overnight at 4 °C with anti-CRISP3 antibody (1:1000). Goat anti-Mouse IgG (H + L) antibody (1:1000, Thermo Fisher, USA) was incubated for 1 h at 37 °C, followed by thorough washing with PBS. The specimens were analyzed under a fluorescence microscope (BX53F, Olympus, Tokyo, Japan).

### 4.9. In Vitro Fertilization Assay

In vitro culture and fertilization of oocytes were performed as previously described [36]. Pig ovaries were procured from a nearby abattoir and expeditiously conveyed to the laboratory within a time frame of 4 h while maintaining a temperature of 37 °C. A sterile syringe retrieved follicular fluid from 3–6 mm follicles and washed it three times with 37 °C DPBS-PVA. Under a microscope, cumulus–oocyte complexes (COCs) were picked out. COCs were washed and placed in 500 μL of mineral oil-covered in vitro maturation media in a four-well plate. (Medium TCM199 supplemented with 10 IU/mL pregnant horse serum gonadotropin, 10 IU/mL human chorionic gonadotropin, 0.1 mg/mL L-cysteine, 10% porcine follicular fluid, 10% fetal bovine serum, and 10 ng/mL epidermal growth factor), the oocytes were cultivated at 38.5 °C in an incubator with 5% CO_2_ for 44–48 h. Cumulus cells were digested with 0.1% hyaluronidase, washed three times in fertilization fluid, and transferred to mineral oil-covered fertilization fluid. Fresh semen was washed thrice, and the sperm concentration reached 1 × 10^6^ sperm/mL with mTBM containing BSA. Fertilization drop with oocytes was incubated for 6 h at 38.5 °C in 5% CO_2_ with capacitated sperms, then transferred to pre-balanced PZM-3 embryo culture medium. The recipient semen was treated with an antibody solution at a final concentration of 2 µg/mL. As a negative control, an equal volume of IgG was also added. After 48 h, fertilization rates were assessed using a 200× microscope (Leica Microsystems, Wetzlar, Germany).

### 4.10. Sperm Motility Assay

Semen samples from the breeding farm were held at 17 °C and quickly transported to the lab for sperm analyses, including motility and morphology. Sperm were resuspended at 5 × 10^6^ sperm/mL after three washings in DPBS with 1 mg/mL BSA. Sperm motility was assessed using a computer-assisted sperm analysis system (CASA; Integrated Sperm Analysis System, ISAS, V1.0; Proiser, Valencia, Spain) with a 37 °C constant temperature table. Following that, droplets measuring 5 µL from each sample were carefully deposited into a disposable counting chamber (Leja, Nieuw-Vennep, The Netherlands) with a depth of 20 µm. These samples were then subjected to analysis utilizing the Computer-Assisted Sperm Analysis (CASA) system. The assessed sperm kinematic parameters encompassed the mean values of total motility (%), curvilinear velocity (µm·s^−1^), straight-line velocity (µm·s^−1^), average path velocity (µm·s^−1^), amplitude of lateral head displacement (µm), whiplash frequency (Hz), wobble (%), linearity (%), mean angular displacement (degree), and straightness (%).

### 4.11. Sperm Capacitation and AR Evaluation

Collected semen samples were washed twice with DPBS to remove sperm and resuspended in modified Tris-buffered medium (mTBM; 11.3 nM NaCl, 0.3 mM KCl, 1 mM CaCl_2_, 0.5 mM pyruvate, 1.1 mM glucose, and 2 mM TRIS) containing 1 mg/mL BSA. CRISP3 antibody or control IgG was also added at 20 μg/mL. Sperm were placed at 37 °C in a 5% CO_2_ incubator for 180 min for capacitation. Capacitated sperm were incubated with 10 μg/mL FITC-conjugated peanut agglutinin (FITC-PNA, Sigma, Marlborough, MA, USA) at 25 °C for 30 min and washed twice with PBS. Sample smears were air-dried on slides and incubated with Hoechst33342 (10 μg/mL, Sigma, Marlborough, MA, USA) for 5 min at room temperature in the dark. Analysis was performed using a fluorescence microscope (BX53F, Olympus, Tokyo, Japan). For each sample, approximately 400 spermatozoa were scored to classify the different patterns as reactive (fluorescence in the acrosome region) or nonreactive (no fluorescence in the acrosome region).

### 4.12. Immunocompetence Assay

Mouse monocytic macrophage leukemia cells (RAW264.7) were cultivated on 6-well plates at a concentration of 5 × 10^5^ cells/mL. The cells were then incubated overnight in a 37 °C incubator with 5% CO_2_ and 100% humidity in DMEM supplemented with 10% FBS to ensure cell adherence. After 3, 5, 10, and 20 µg/mL of CRISP3 protein processing for 30 min, 1 µg/mL lipopolysaccharide (LPS) (Solarbio, Beijing, China) was added to induce inflammation. To avoid the effect of the protein itself on the cells, only 3, 5, 10, and 20 μg/mL CRISP3 protein groups were added. CRISP3 protein and LPS were dissolved in PBS, and the cells were harvested 6 h later for qRT-PCR and Western blot analysis. All experiments were repeated thrice.

### 4.13. Statistical Analysis

All statistical analyses used SPSS (version 18.0; IBM Corp., Armonk, NY, USA) and GraphPad Prism 8 (version 8.0.2; La Jolla, CA, USA). All data are shown as mean ± standard error. The relationship between CRISP3 protein content and reproductive factors was examined using Pearson’s correlation analysis. Statistical differences among groups were determined using the student’s *t*-test and ANOVA. Statistical significance was attributed to those *p* values < 0.05.

## 5. Conclusions

In our study, we found that the sperm CRISP3 protein content was significantly positively correlated with the reproductive performance of boars. The CRISP3 protein was abundant in the bulbourethral glands of adult boars, distributed in the post-acrosomal region and tail of the sperm head, and it was relocalized to the upper middle part of the tail after capacitation. We also found that the CRISP3 plays an anti-inflammatory role by inhibiting LPS-induced production of inflammatory factors in RAW264.7 cells. This study revealed that CRISP3 protein regulates the immune function of the female reproductive tract and provides new clues for CRISP3 protein as a marker of male fertility.

## Figures and Tables

**Table 1:** Relevance analysis of parameters associated with sperm motility between the negative control group and the group treated with anti-CRISP3 antibodies.

Motility Parameters	Negative Control	Anti-CRISP3 Antibody	*p* Value
TM (%)	76.75 ± 2.80	76.06 ± 1.32	0.836
VSL (μm/s)	28.57 ± 0.81	28.96 ± 0.64	0.722
VCL (μm/s)	48.79 ± 1.70	49.60 ± 1.31	0.725
VAP (μm/s)	34.97 ± 0.98	35.07 ± 0.92	0.944
ALH (μm)	14.48 ± 0.41	14.53 ± 0.38	0.942
WOB (%)	68.00 ± 2.00	64.00 ± 1.00	0.137
BCF (Hz)	0.74 ± 0.01	0.73 ± 0.01	0.624
LIN (%)	58.00 ± 1.00	58.00 ± 0.00	0.519
MAD (°)	114.45 ± 2.71	103.81 ± 4.26	0.103
STR (%)	82.00 ± 1.00	83.00 ± 1.00	0.482

TM, total motility; PM, progressive motility; VCL, curvilinear velocity; VSL, straight line velocity; VAP, average path velocity; ALH, mean amplitude of head lateral displacement; WOB, wobble; BCF, beat cross frequency; LIN, linearity; MAD, mean angular displacement; STR, straightness; Negative control meant that sperm were exposed to IgG; Values are expressed as mean ± standard error of the mean; The experiment consisted of three replicates.

**Table 2:** Effect of anti-CRISP3 antibodies on the cleavage rate of in vitro fertilization.

Groups	No. of Oocytes	No. of Cleaved	Cleavage Rate %
Control	324	192	59.53 ± 2.54 ^A^
IgG	273	157	57.50 ± 2.03 ^A^
Anti-CRISP3	279	153	55.04 ± 0.89 ^A^

Values are expressed as mean ± standard error of the mean; the experiment included six replicates. Different letters in the same column indicate significant differences (*p* < 0.05).

## Data Availability

The data presented in this study are available on request from the corresponding author.

## Supplementary Materials

The following supporting information can be downloaded at: https://www.mdpi.com/article/10.3390/ijms25042264/s1.

## References

[1] Druart X., de Graaf S. (2018). Seminal plasma proteomes and sperm fertility. Anim. Reprod. Sci..

[2] Shang X., Shen C., Liu J., Tang L., Zhang H., Wang Y., Wu W., Chi J., Zhuang H., Fei J. (2018). Serine protease PRSS55 is crucial for male mouse fertility via affecting sperm migration and sperm-egg binding. Cell Mol. Life Sci..

[3] Roca J., Perez-Patino C., Barranco I., Padilla L.C., Martinez E.A., Rodriguez-Martinez H., Parrilla I. (2020). Proteomics in fresh and preserved pig semen: Recent achievements and future challenges. Theriogenology.

[4] Taylor J.F., Schnabel R.D., Sutovsky P. (2018). Identification of genomic variants causing sperm abnormalities and reduced male fertility. Anim. Reprod. Sci..

[5] Jalkanen J., Huhtaniemi I., Poutanen M. (2005). Mouse cysteine-rich secretory protein 4 (CRISP4): A member of the Crisp family exclusively expressed in the epididymis in an androgen-dependent manner. Biol. Reprod..

[6] Magdaleno L., Gasset M., Varea J., Schambony A.M., Urbanke C., Raida M., Topfer-Petersen E., Calvete J.J. (1997). Biochemical and conformational characterisation of HSP-3, a stallion seminal plasma protein of the cysteine-rich secretory protein (CRISP) family. FEBS Lett..

[7] Udby L., Lundwall A., Johnsen A.H., Fernlund P., Valtonen-Andre C., Blom A.M., Lilja H., Borregaard N., Kjeldsen L., Bjartell A. (2005). beta-Microseminoprotein binds CRISP-3 in human seminal plasma. Biochem. Biophys. Res. Commun..

[8] Vadnais M.L., Foster D.N., Roberts K.P. (2008). Molecular cloning and expression of the CRISP family of proteins in the boar. Biol. Reprod..

[9] Hu J., Merriner D.J., O’Connor A.E., Houston B.J., Furic L., Hedger M.P., O’Bryan M.K. (2018). Epididymal cysteine-rich secretory proteins are required for epididymal sperm maturation and optimal sperm function. Mol. Hum. Reprod..

[10] Lim S., Kierzek M., O’Connor A.E., Brenker C., Merriner D.J., Okuda H., Volpert M., Gaikwad A., Bianco D., Potter D. (2019). CRISP2 Is a Regulator of Multiple Aspects of Sperm Function and Male Fertility. Endocrinology.

[11] Ernesto J.I., Weigel M.M., Battistone M.A., Vasen G., Martinez-Lopez P., Orta G., Figueiras-Fierro D., De la Vega-Beltran J.L., Moreno I.A., Guidobaldi H.A. (2015). CRISP1 as a novel CatSper regulator that modulates sperm motility and orientation during fertilization. J. Cell Biol..

[12] Sun X.H., Zhu Y.Y., Wang L., Liu H.L., Ling Y., Li Z.L., Sun L.B. (2017). The Catsper channel and its roles in male fertility: A systematic review. Reprod. Biol. Endocrinol..

[13] Gholami D., Amirmahani F., Yazdi R.S., Hasheminia T., Teimori H. (2021). MiR-182-5p, MiR-192-5p, and MiR-493-5p Constitute a Regulatory Network with CRISP3 in Seminal Plasma Fluid of Teratozoospermia Patients. Reprod. Sci..

[14] Heidary Z., Zaki-Dizaji M., Saliminejad K., Khorramkhorshid H.R. (2019). Expression Analysis of the CRISP2, CATSPER1, PATE1 and SEMG1 in the Sperm of Men with Idiopathic Asthenozoospermia. J. Reprod. Infertil..

[15] Carvajal G., Brukman N.G., Weigel M.M., Battistone M.A., Guazzone V.A., Ikawa M., Haruhiko M., Lustig L., Breton S., Cuasnicu P.S. (2018). Impaired male fertility and abnormal epididymal epithelium differentiation in mice lacking CRISP1 and CRISP4. Sci. Rep..

[16] Curci L., Brukman N.G., Weigel M.M., Rojo D., Carvajal G., Sulzyk V., Gonzalez S.N., Rubinstein M., Da R.V., Cuasnicu P.S. (2020). Functional redundancy and compensation: Deletion of multiple murine Crisp genes reveals their essential role for male fertility. FASEB J..

[17] Haendler B., Kratzschmar J., Theuring F., Schleuning W.D. (1993). Transcripts for cysteine-rich secretory protein-1 (CRISP-1; DE/AEG) and the novel related CRISP-3 are expressed under androgen control in the mouse salivary gland. Endocrinology.

[18] Kjeldsen L., Cowland J.B., Johnsen A.H., Borregaard N. (1996). SGP28, a novel matrix glycoprotein in specific granules of human neutrophils with similarity to a human testis-specific gene product and a rodent sperm-coating glycoprotein. FEBS Lett..

[19] Kratzschmar J., Haendler B., Eberspaecher U., Roosterman D., Donner P., Schleuning W.D. (1996). The human cysteine-rich secretory protein (CRISP) family. Primary structure and tissue distribution of CRISP-1, CRISP-2 and CRISP-3. Eur. J. Biochem..

[20] Evans J., D’Sylva R., Volpert M., Jamsai D., Merriner D.J., Nie G., Salamonsen L.A., O’Bryan M.K. (2015). Endometrial CRISP3 is regulated throughout the mouse estrous and human menstrual cycle and facilitates adhesion and proliferation of endometrial epithelial cells. Biol. Reprod..

[21] Schambony A., Gentzel M., Wolfes H., Raida M., Neumann U., Topfer-Petersen E. (1998). Equine CRISP-3: Primary structure and expression in the male genital tract. Biochim. Biophys. Acta.

[22] Da R.V., Munoz M.W., Battistone M.A., Brukman N.G., Carvajal G., Curci L., Gomez-ElIas M.D., Cohen D.B., Cuasnicu P.S. (2015). From the epididymis to the egg: Participation of CRISP proteins in mammalian fertilization. Asian J. Androl..

[23] Lee U., Nam Y.R., Ye J.S., Lee K.J., Kim N., Joo C.H. (2014). Cysteine-rich secretory protein 3 inhibits hepatitis C virus at the initial phase of infection. Biochem. Biophys. Res. Commun..

[24] Doty A., Buhi W.C., Benson S., Scoggin K.E., Pozor M., Macpherson M., Mutz M., Troedsson M.H. (2011). Equine CRISP3 modulates interaction between spermatozoa and polymorphonuclear neutrophils. Biol. Reprod..

[25] Chen Y., Wei H., Liu Y., Gao F., Chen Z., Wang P., Li L., Zhang S. (2020). Identification of new protein biomarkers associated with the boar fertility using iTRAQ-based quantitative proteomic analysis. Int. J. Biol. Macromol..

[26] Usuga A., Rojano B.A., Restrepo G. (2018). Association of the cysteine-rich secretory protein-3 (CRISP-3) and some of its polymorphisms with the quality of cryopreserved stallion semen. Reprod. Fertil. Dev..

[27] Restrepo G., Rojano B., Usuga A. (2019). Relationship of cysteine-rich secretory protein-3 gene and protein with semen quality in stallions. Reprod. Domest. Anim..

[28] Mistretta V.I., Cavalier E., Collette J., Chapelle J.P. (2009). Production of monoclonal antibodies. Rev. Med. Liege.

[29] Song C.Y., Gao B., Wu H., Wang X.Y., Zhou H.Y., Wang S.Z., Li B.C., Chen G.H., Mao J.D. (2011). Spatial and temporal gene expression of Fn-type II and cysteine-rich secretory proteins in the reproductive tracts and ejaculated sperm of Chinese Meishan pigs. Reprod. Domest. Anim..

[30] Anklesaria J.H., Pandya R.R., Pathak B.R., Mahale S.D. (2016). Purification and characterization of CRISP-3 from human seminal plasma and its real-time binding kinetics with PSP94. J. Chromatogr. B.

[31] Weigel M.M., Carvajal G., Curci L., Gonzalez S.N., Cuasnicu P.S. (2019). Relevance of CRISP proteins for epididymal physiology, fertilization, and fertility. Andrology.

[32] Gonzalez S.N., Sulzyk V., Weigel M.M., Cuasnicu P.S. (2021). Cysteine-Rich Secretory Proteins (CRISP) are Key Players in Mammalian Fertilization and Fertility. Front. Cell Dev. Biol..

[33] Liu H., Yu H., Gu Y., Xin A., Zhang Y., Diao H., Lin D. (2013). Human beta-defensin DEFB126 is capable of inhibiting LPS-mediated inflammation. Appl. Microbiol. Biotechnol..

[34] Rhule A., Navarro S., Smith J.R., Shepherd D.M. (2006). Panax notoginseng attenuates LPS-induced pro-inflammatory mediators in RAW264.7 cells. J. Ethnopharmacol..

[35] Hnasko R.M., McGarvey J.A. (2015). Affinity Purification of Antibodies. Methods Mol. Biol..

[36] Li J., Wei H., Li Y., Li Q., Li N. (2012). Identification of a suitable endogenous control gene in porcine blastocysts for use in quantitative PCR analysis of microRNAs. Sci. China Life Sci..

