# Supplementary material for: Semen Protein CRISP3 Promotes Reproductive Performance of Boars through Immunomodulation
Source: Int J Mol Sci. 2024 Feb 14;25(4):2264. doi: 10.3390/ijms25042264 (PMC10889302; doi:10.3390/ijms25042264)
Supplement: Supplementary file 1 [file ijms-25-02264-s001.zip › ijms-2864053-supplementary.pdf]

Supplementary material

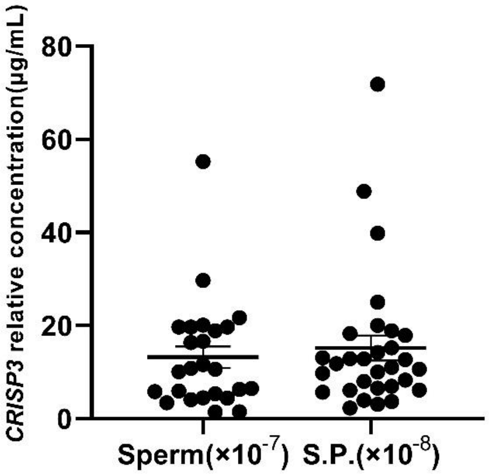

Figure S1. Boar sperm and seminal plasma protein CRISP3 relative content determination.

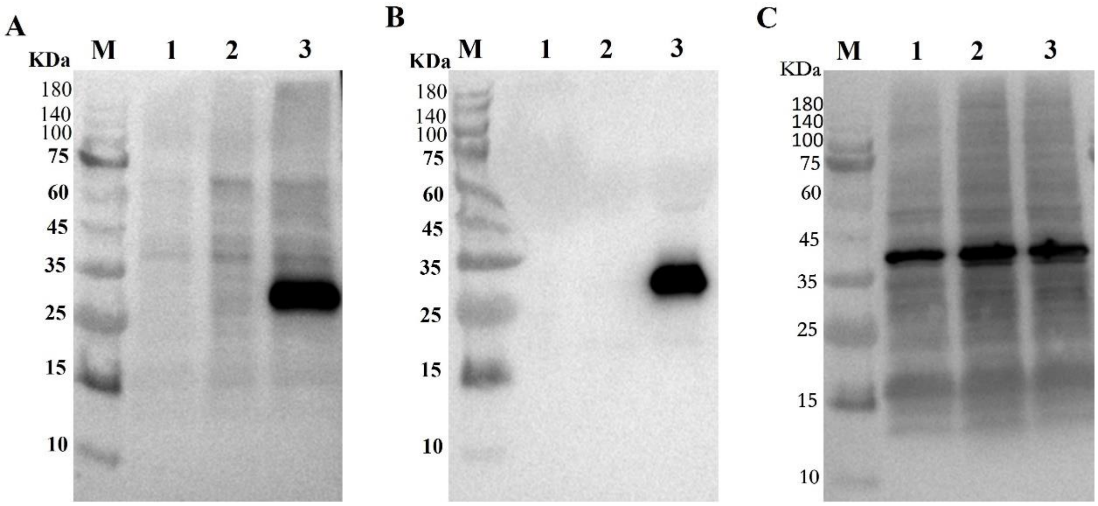

Figure S2. Identification of CRISP3 protein expression in CHO-K1 cells and supernatant. (A-B) Western blot analysis of His tag protein in CHO-K1 cell lysate and supernatant, respectively. (C) CHO-K1 Western blot analysis of GAPDH internal reference protein in cell lysates. Lane 1: untreated CHO-K1 cells; Lane 2: CHO-K1 cells transfected with empty vector; Lane 3: CHO-K1 cells carrying the CRISP3 gene vector were transfected.

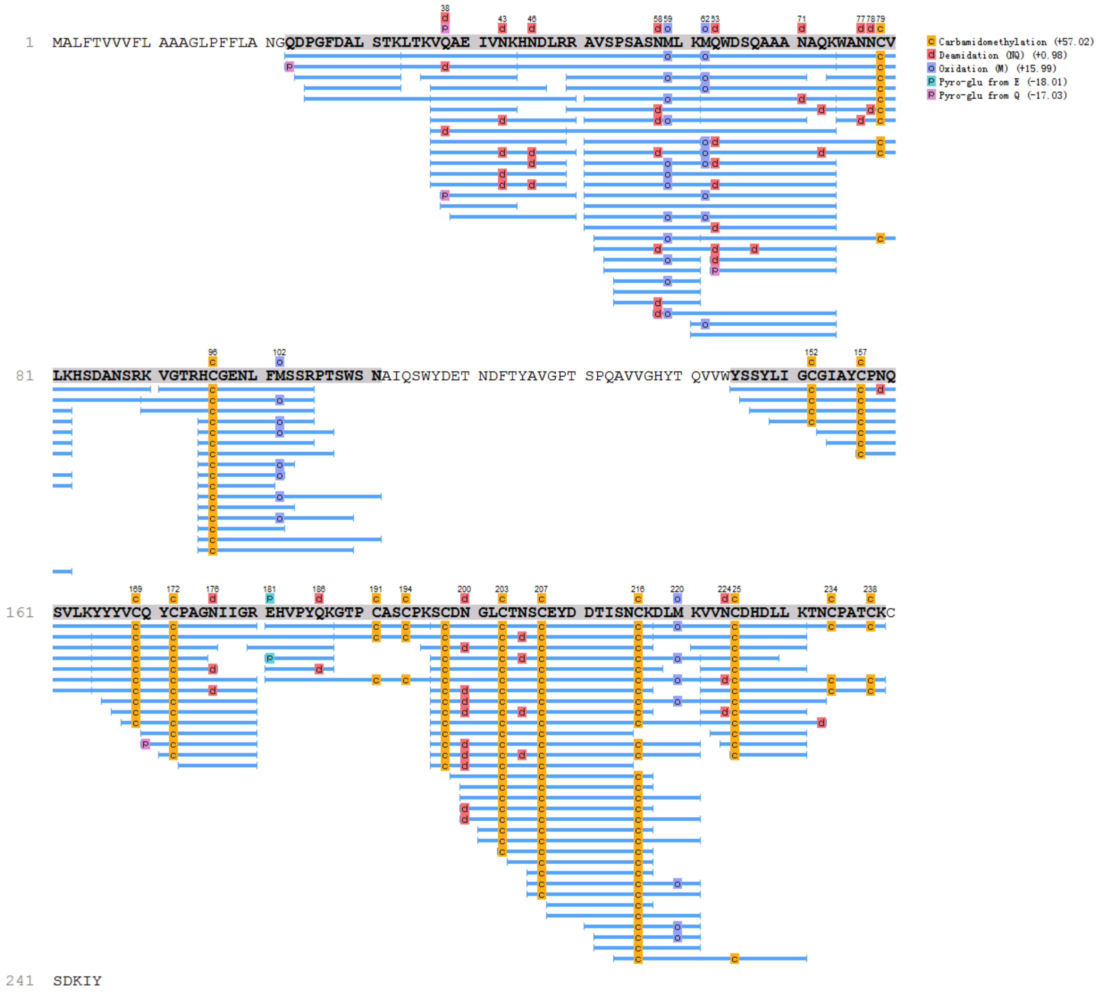

Figure S3. Peptide coverage map of the porcine CRISP3 recombinant protein by LC-MS/MS mass spectrometry.

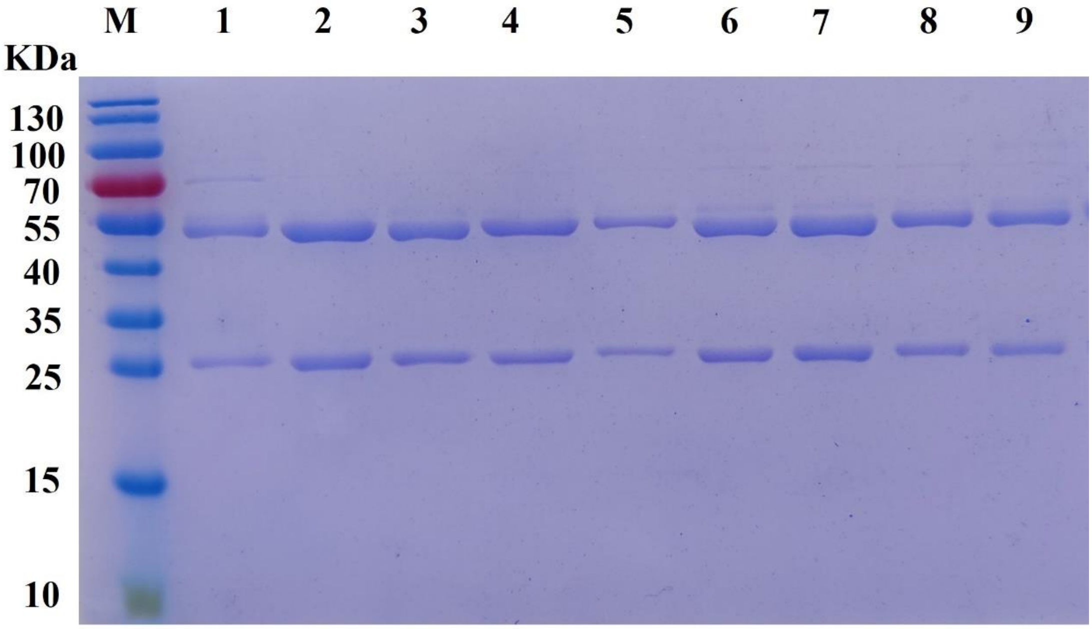

Figure S4. Identification by SDS-PAGE of CRISP3 monoclonal antibodies. M: 180 KDa protein marker; Lanes 1-9: Nine monoclonal antibodies.

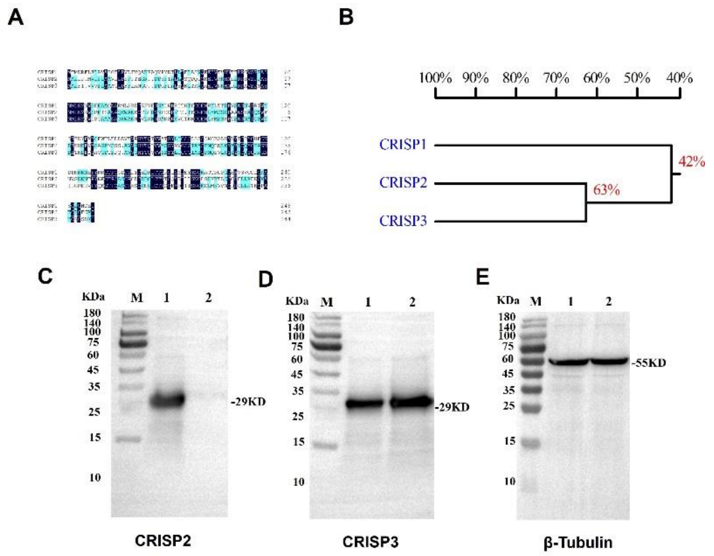

Figure S5. Comparison of amino acid homology among porcine CRISP1, CRISP2, and CRISP3 and specificity identification of antibodies against CRISP3 in boars. (A) Amino acid sequence alignment of porcine CRISP1, CRISP2, and CRISP3. (B) Homologous tree of porcine CRISP1, CRISP2, and CRISP3. (C) Western blot analysis of boar testis and bulbourethral gland bands recognized by anti-CRISP2 antibody. M: Protein Marker; 1: Boar testis; 2: Boar bulbourethral gland. (D) Western blot analysis of boar testis and bulbourethral gland bands recognized by anti-CRISP3 antibody. M: Protein Marker; 1: Boar testis; 2: Boar bulbourethral gland. (E) Western blot analysis of β-tubulin reference proteins in boar testis and bulbourethral glands. M: Protein Marker; 1: Boar testis; 2: Boar bulbourethral gland.

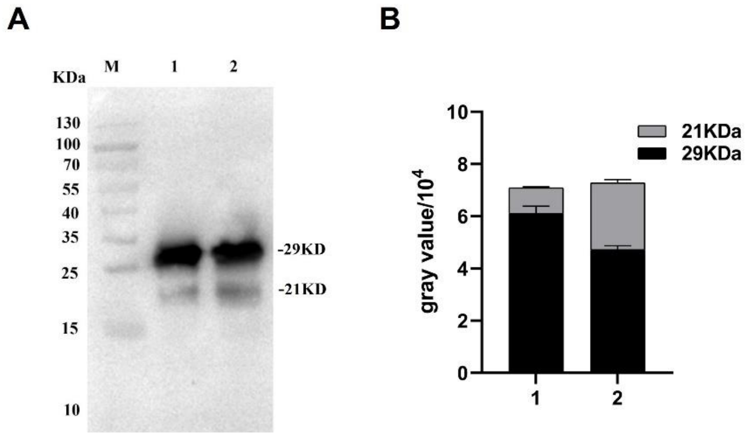

Figure S6. Western blot analysis of glycosylation of porcine CRISP3 recombinant protein, (A) M: protein Marker; 1: without glycosidase treatment of pig CRISP3 recombinant protein; 2: Glycosidase-treated porcine CRISP3 recombinant protein. (B) Western blot analysis of CRISP3 protein expression before and after glycosidase F treatment was performed using Image J software; 1: lane 1; 2: lane 2. The bar graphs show the gray values at 29 KDa and 21 KDa in lane 1 and 2, respectively.

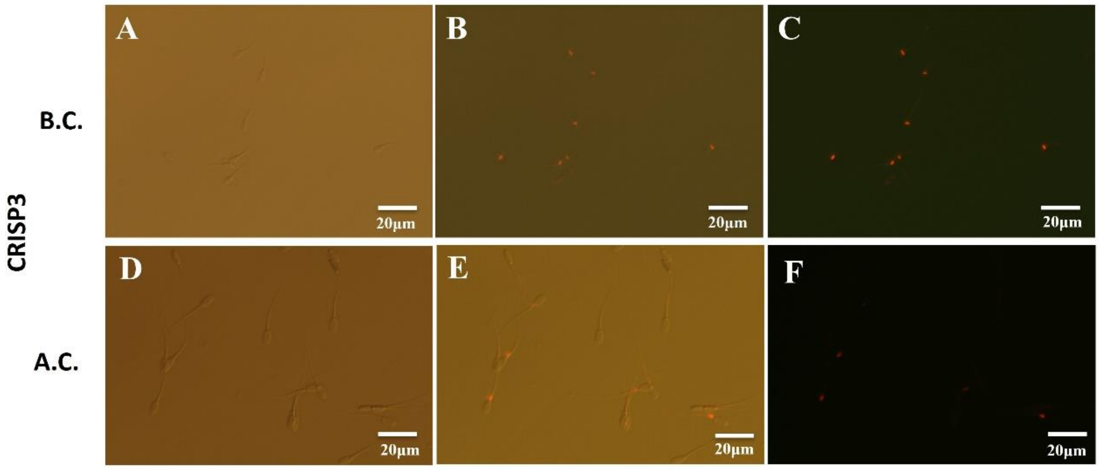

Figure S7. Immunofluorescent staining of CRISP3 in sperm before and after capacitation. (A-C) Representative images of the immunofluorescent staining of CRISP3 in sperm before capacitation; (A) images taken under light microscope, (B) images taken under fluorescent scope; (C) merged images. (D-F) Representative images of the immunofluorescent staining of CRISP3 in sperm after capacitation; (D) images taken under light microscope, (E) images taken under fluorescent scope; (F) merged images. B.C., before capacitation; A.C., after capacitation. The arrow indicates the distribution of the target proteins.

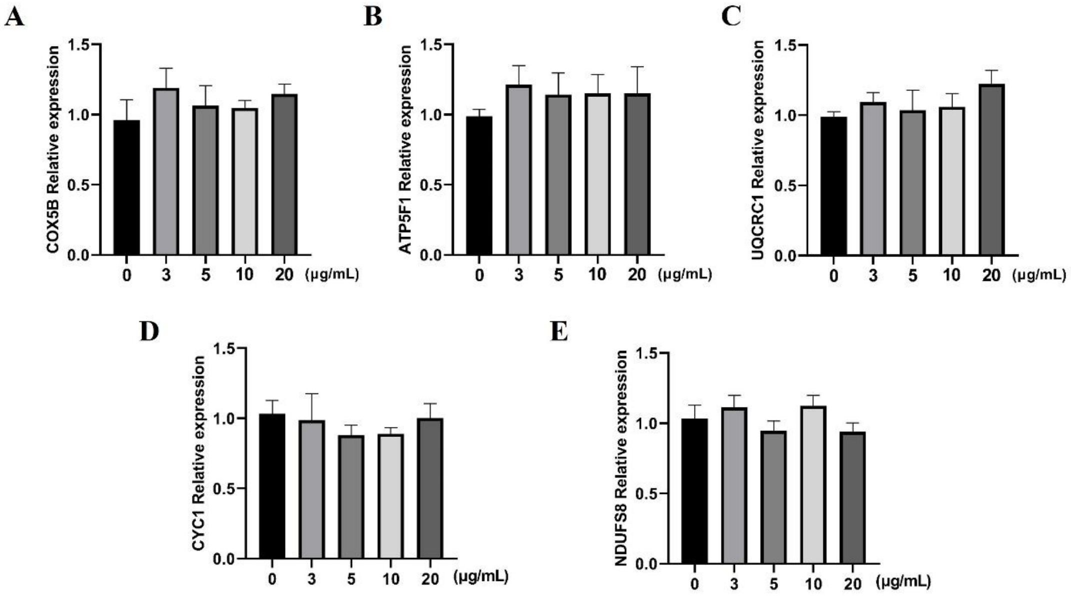

Figure S8. Effects of CRISP3 recombinant protein on COX5B, ATP5F1 and UQCRC1 mRNA expression in sperm. (A) COX5B mRNA expression level; (B) ATP5F1 mRNA expression level; (C) UQCRC1 mRNA expression level. (D) CYC1 mRNA expression level. (E) NDUFS8 mRNA expression level. GAPDH was included in the standardization of all samples as an internal reference gene, and the data were expressed as the mean ± SEM of three independent experiments.

Table S1. RT-PCR and qRT-PCR primers

	Genes	Forward (5' to 3')	Reverse (5' to 3')	Product size (bp)
RT-PCR	*β-actin*	CCAAAGCCAACCGTGAGA	CTCGTTGCCGATGGTGAT	425
	CRISP3	CCCCACTGAAGAGTAACCCC	GACCGCAGTCCAGGATTCAT	143
qRT-PCR	*GAPDH*	GAGATCCCGCCAACATCAAAT	GTTCACGCCCATCACAAACAT	170
	CRISP3	CCCCACTGAAGAGTAACCCC	GACCGCAGTCCAGGATTCAT	168
	IL-1α	AAGCAGCCTTATTTCGGGAG	TATCATATGTCGGGGTGGCT	187
	IL-1β	GCAACTGTTCCTGAACTCAACT	ATCTTTTGGGGTCCGTCAACT	89
	IL-6	GGCCTTCCCTACTTCACAAG	GCAAGTGCATCATCGTTGTT	183
	GAPDH	TCCAAGGAGTAAGAAACCCTGGAC	GTTATTATGGGGGTCTGGGATGG	128
	COX5B	TATGGCATCTGGAGGTGGTG	CAGCCCACTATCCGCTTGTT	183
	ATP5F1	CCAAGACCGTTCTGTGGACG	TCCTTGTTGCCTGCAGTACC	127
	UQCRC1	CGCCTTGTTCACTTTACGCA	GAGACCTCAGCAAGGCCG	144
	CYC1	CTGAACCCGAACACGACCAT	AGTTTCCGGCTCTTGAGGAC	124
	NDUFS8	CTCTTGAATCCAGCTCGGCG	TCCGCGCTGACTCTTCATAG	141
